# Hierarchically Structured Ti-TiO_2_ Membranes Fabricated by Femtosecond Laser Ablation and Atomic Layer Deposition for Enhanced Photoelectrochemical Water Splitting

**DOI:** 10.1021/acsami.5c07488

**Published:** 2025-07-21

**Authors:** Andrii Lys, Iaroslav Gnilitskyi, Emerson Coy, Mariusz Jancelewicz, Mikhael Bechelany, Igor Iatsunskyi

**Affiliations:** † NanoBioMedical Centre, 49562 Adam Mickiewicz University, 3, Wszechnicy Piastowskiej Str., 61-614 Poznan, Poland; ‡ “NoviNano” Lab LLC, Pasternaka, 5, 79015 Lviv, Ukraine; § Department of Applied Physics and Nanomaterials Science, 226328 Lviv Polytechnic National University, 12, Bandery Str., 79013 Lviv, Ukraine; ∥ Institut Européen des Membranes, IEM, UMR 5635, University of Montpellier, ENSCM, CNRS, 34090 Montpellier, France; ⊥ Functional Materials Group, Gulf University for Science and Technology (GUST), Mubarak Al-Abdullah 32093, Kuwait

**Keywords:** photoelectrochemical water splitting, hierarchical TiO_2_, titanium membrane, femtosecond laser ablation, atomic layer deposition, binder-free photoanode, solar hydrogen production

## Abstract

Efficient and scalable photoelectrodes are essential for advancing solar-driven hydrogen production via photoelectrochemical (PEC) water splitting. This work presents a novel binder-free Ti-TiO_2_ membrane photoanode engineered by the synergy of femtosecond laser ablation and atomic layer deposition (ALD). Laser processing produced a highly ordered array of micro pyramids on titanium foil, significantly increasing the surface area and light-trapping capability. Subsequent ALD of TiO_2_ (10 and 100 nm) yielded conformal coatings with tunable crystallinity. Among the tested configurations, the 100 nm TiO_2_ layer showed superior performance, attributed to its enhanced crystallinity, optical absorption, and charge transport properties. The optimized membrane achieved a photocurrent density of ∼27 μA cm^–2^ at 1.4 V vs NHE, an IPCE (Incident photon to current conversion efficiency) of ∼31% at 275 nm, and a 3-fold increase in ABPE (Applied Bias Photon-to-current Efficiency) compared to the uncoated sample. This strategy presents a scalable and reproducible approach to high-performance, binder-free photoanodes for solar hydrogen production.

## Graphical abstract

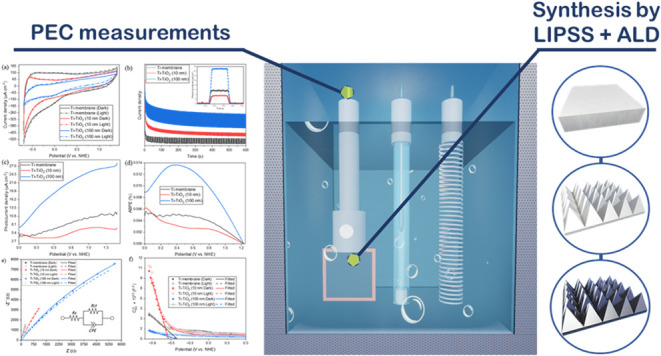

## 1 Introduction

Over the last century, the significant consumption of fossil fuels, technological advancements, and the Industrial Revolution have contributed to a nearly 50% increase in atmospheric CO_2_ concentration.^1^ This rise, in turn, is the driving force behind climate change, global warming, environmental problems, and deterioration of health conditions.^2^ While hydrogen (H_2_) is often viewed as a clean, high-energy fuel with strong potential for decarbonization, its current production methods are mainly unsustainable,^3^ because over 90% of global hydrogen is still derived from fossil fuel-based technologies, leading to considerable CO_2_ emissions.^4^ Among all the available alternative technologies, photoelectrochemical (PEC) water splitting is a promising approach for generating clean hydrogen. This method uses semiconductor materials to capture sunlight, producing electron–hole pairs that facilitate the dissociation of water into hydrogen and oxygen.^5^

Titanium dioxide (TiO_2_) has attracted significant attention as a photoanode material due to its affordability, chemical stability, and nontoxic nature.^6^ Our previous work demonstrated that atomic layer deposition (ALD) allows precise control over the thickness of metal oxide layers, including TiO_2_.^7–10^ However, its wide band gap (∼3.2 eV) limits its absorption to the UV region, hindering efficient charge separation and negatively impacting the PEC performance. As a result, its overall efficiency remains unsatisfactory.^11^ To overcome these challenges, several solutions have been explored, including the incorporation of noble metals,^12,13^ dopant engineering,^14,15^ heterostructure formation,^13,16^ and morphological modifications.^17,18^

In this context, the application of femtosecond laser-based surface engineering has gained growing interest due to its precision, speed, high reproducibility, and contactless characteristics. It provides a powerful and versatile tool for tailoring surface properties of materials and significantly enhancing their functionality.^19^ Laser-processed functional surface structures (LPFSS) have been extensively explored for diverse applications in surface texturing, wettability control, and functional coatings.^20,21^

Some laser-structured surfaces, referred to as hierarchical structures, have complex shapes and large surface areas that enhance photocatalytic properties.^22^ Zhou et al. demonstrated that microstructured arrays alter optical properties and significantly impact impedance, resulting in a considerable reduction in impedance due to morphology-induced changes.^23^ When comparing laser treatment with the widely used electrochemical machining (ECM) technique, several key disadvantages of ECM become evident. The factors to consider encompass corrosive characteristics, reliance on the electrical properties of the metal, significant overcut, and scalability constraints for industrial applications.^24^ On the other hand, laser-based micromachining has emerged as a highly effective and dependable method for extensive surface treatment and modification. It provides exceptional precision at the microscopic scale, operates without contact, and offers remarkable versatility for working with challenging materials.^25,26^

Several previous studies have explored hierarchical structuring of titanium-based photoelectrodes to enhance light absorption and charge separation. In particular, Liang et al.^27^ combined femtosecond laser processing with anodization to fabricate TiO_2–*x*_ hierarchical nanotube arrays containing oxygen vacancies, which exhibited significantly enhanced photocatalytic activity due to bandgap narrowing and defect-mediated charge transfer. Nonetheless, the introduction of elevated concentrations of oxygen vacancies could potentially undermine long-term stability in photoelectrochemical processes.

In this study, ALD and laser-processed functional surface structures (hierarchical structures) were integrated to fabricate a novel titanium­(Ti)-based membrane. The membrane was first structured via femtosecond laser ablation, forming a micrometric array of titanium pyramids, followed by ALD deposition of TiO_2_ to introduce a photoactive layer. The resulting highly regular Ti-TiO_2_ membrane functions as a photoanode for electrochemical water splitting, aiming to develop a new, binder-free electrode for sustainable hydrogen production. In contrast to conventional Ti/TiO_2_ systems, this work introduces a scalable laser-assisted ALD integration strategy that enables precise conformal coating over complex hierarchical structures, providing a versatile platform for future photovoltaic electrochemical (PEC) device optimization. We present a comprehensive physicochemical characterization of the fabricated composites, employing Raman spectroscopy, contact-angle analysis, scanning electron microscopy (SEM), X-ray diffraction (XRD), X-ray photoelectron spectroscopy (XPS), and ultraviolet–visible (UV–vis) spectroscopy. Additionally, we perform a series of photoelectrochemical tests, including cyclic voltammetry (CV), linear sweep voltammetry (LSV), chronoamperometry (CA), electrochemical impedance spectroscopy (EIS), Mott–Schottky analysis, and incident photon-to-current efficiency (IPCE) measurements.

## 2 Experimental Section

### 2.1 Materials and Synthesis

Titanium tetrachloride (TiCl_4_, 99.9% trace metals basis, ReagentPlus, Sigma-Aldrich) and sodium sulfate (Na_2_SO_4_, ≥ 99.0%, ACS reagent grade, anhydrous, Sigma-Aldrich) were used without further purification. Deionized water with a resistivity of 18.2 MΩ·cm was obtained from a Milli-Q system. High-purity argon gas (Ar, 5.0, 99.999%, Linde HiQ, UN 1006) was used to purge the electrolyte solution before PEC measurements. The Ti membrane was fabricated using a Pharos P20 fs laser system from LightConversion (Lithuania). This system delivers a p-polarized (PP) laser beam with excellent beam quality (M^2^ < 1.2), a beam diameter of 2.5 mm, a central wavelength of 1030 nm, a pulse duration of 266 fs, a maximum average power of 20 W, and a maximum pulse repetition frequency of 1 MHz. The average laser power and pulse repetition rate were precisely controlled using the Pharos control software. The generated laser beam was enlarged by passing through an expander and then forwarded to a galvanometric scanning head ExcelliScan (Scan Lab, Germany). Quadratic Ti coupons measuring 1 cm × 1 cm were cut from commercially pure titanium (>99% Ti, grade 1). The Ti samples were polished with abrasive papers to increase fineness and remove significant surface imperfections before undergoing laser micromachining. Square pyramid structures were fabricated via raster scanning at an incident angle (θ) of 0°, employing a laser fluence of 1.3 J/cm^2^, a repetition rate of 1 MHz, a scanning speed of 3 m/s, a line overlap of 83%, and delivering 300 pulses per spot (PPS), under ambient atmospheric conditions. The chosen fluence, repetition rate, and scanning speed yielded the most uniform and reproducible pyramid arrays. Lower fluence levels (<1.0 J/cm^2^) resulted in insufficient ablation and poorly defined surface features, compromising the formation of uniform microstructures, while higher fluences (>1.5 J/cm^2^) led to excessive melting and random redeposition. The selected parameters ensured a balance between material removal and morphological definition. The sample-carrying stage moved along the *x*-axis, enabling precise laser spot scanning. Upon completion of each scanline, the stage returned to its initial position and shifted incrementally along the *y*-axis to initiate the subsequent scanline. An electro-optic modulator-based beam shutter was utilized to selectively block the laser beam when traversing designated pyramid locations, thereby ensuring these regions remained unablated. This overscanning procedure was repeated multiple times to attain the desired final pillar height. Additionally, after each overscan cycle, the z-position of the stage was adjusted to maintain the laser focal point on the sample surface. Following the formation of the Ti membrane, two different TiO_2_ layers were deposited using ALD at 150 °C with TiCl_4_ and water as precursors. The protocol for depositing thin TiO_2_ layers on nano- and microstructures has been previously optimized and developed.^28,29^ The first deposition consisted of 250 ALD cycles, yielding a TiO_2_ layer of approximately 10 nm, while the second deposition comprised 2500 ALD cycles, resulting in a TiO_2_ thickness of approximately 100 nm. The typical growth rate was 0.4 Å/cycle. Simultaneously, a metal oxide layer was grown on a planar Si surface to confirm the thickness using ellipsometry measurements. A schematic representation of the sample preparation procedure is presented in Figure 1.

**Figure 1:**
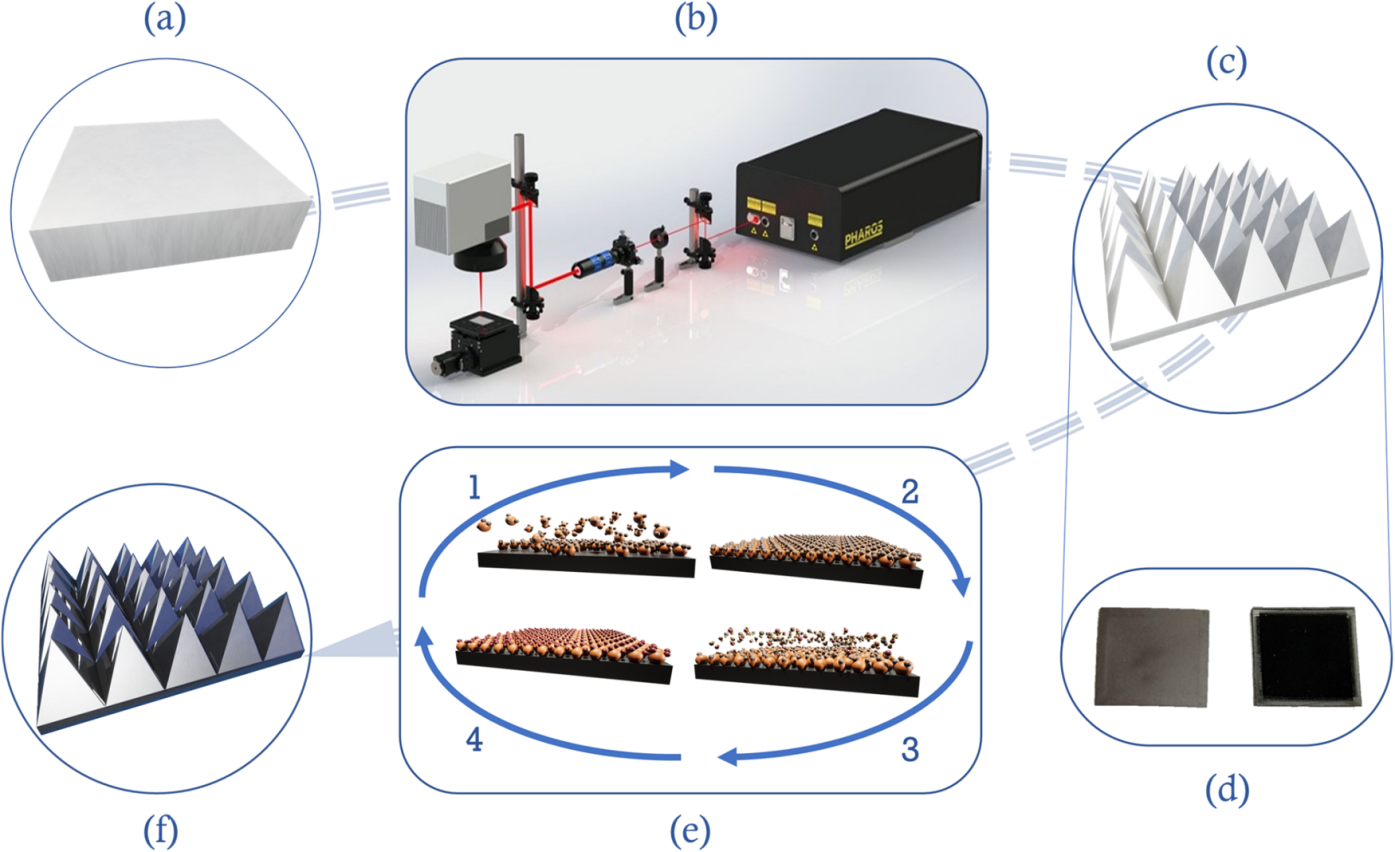
Schematic representation of the production cycle of the Ti-TiO_2_ membrane: (a) Ti substrate, (b) laser setup for membrane formation, (c) Ti membrane, (d) photographs of nonprocessed and laser-processed Ti surfaces, (e) ALD TiO_2_ deposition process (1. precursor pulse, 2. precursor purge, 3. oxidizer pulse, 4. oxidizer purge), and (f) final Ti-TiO_2_ membrane.

### 2.2 Characterization

The structure of the produced nanocomposites and individual materials was investigated using scanning electron microscopy (SEM) (JEOL JSM7001F). Raman spectra were recorded with a Renishaw micro-Raman spectrometer equipped with a confocal microscope and a 633 nm excitation laser. X-ray photoelectron spectroscopy (XPS) with a monochromatic X-ray source (Al–Kα; hυ = 1486.6 eV) was performed to determine the surface elemental composition. The optical properties, including absorbance and photoluminescence, were analyzed using an Ocean Optics QE65Pro spectrophotometer. Diffuse reflectance measurements were conducted using an Ocean Optics QE PRO spectrometer coupled with an integrating sphere. A series of electrochemical tests was performed using a Gamry Reference 620 potentiostat.

### 2.3 Photoelectrochemical Tests

The PEC properties of the produced Ti-TiO_2_ membranes were evaluated using a standard three-electrode system. The membranes served as the working electrodes, a platinum wire was the counter electrode, and an Ag/AgCl (3 M KCl) electrode was used as the reference electrode. All measurements were conducted at room temperature. A 0.5 M Na_2_SO_4_ electrolyte solution with pH ∼7 was diaurated by purging with argon (Ar) gas for 30 min before and during all experiments to remove dissolved oxygen. The samples were irradiated with a 300 W xenon lamp, adjusted to a power density of 100 mW/cm^2^. IPCE measurements were performed using a monochromator to control the wavelength of incident light. CA measurements were conducted under continuous illumination at a bias potential of 1.23 V vs the normal hydrogen electrode (NHE) to evaluate the photocurrent stability. Electrochemical impedance spectroscopy (EIS) was conducted both in the dark and under illumination, within a frequency range of 0.1 Hz to 5 MHz, using an amplitude of 10 mV and a bias potential of 1.23 V vs NHE. The Mott–Schottky analysis was performed at a fixed frequency of 1 kHz, over a potential range of −0.8 to 1.23 V vs NHE. The photoconversion efficiency, specifically the applied bias photon-to-current conversion efficiency (ABPE, %) of the fabricated electrodes, was calculated.^30^

1 $$ABPE\left(\%\right)=\frac{\left[I_{ph}\left(\frac{mA}{{cm}^{2}}\right)\times \left(1.23-\left|V_{RHE}\right|\right)\left(V\right)\right]}{\left[P_{ls}\left(\frac{mW}{{cm}^{2}}\right)\right]}\times 100\left(\%\right)$$

where *I*_ph_ is the measured photocurrent density, *P*_ls_ is the intensity of the light source, and *V*_RHE_ is the applied potential of the PEC cell, recalculated relative to NHE using the Nernst equation:^30^

2 $$V_{\mathrm{RHE}}=V_{\mathrm{app}}+V_{\mathrm{Ag}/\mathrm{AgCl}}^{0}+0.059\times \mathrm{pH}$$

where *V*_app_ is the potential measured against the Ag/AgCl reference electrode, *V*_Ag/AgCl_^0^ is the standard electrode potential of Ag/AgCl electrode (0.1976 V).

## 3 Results and Discussion

### 3.1 The Morphology and Structural Characterization

The morphology of the Ti membrane, the Ti membrane with a 10 nm TiO_2_ layer deposited by ALD (Ti-TiO_2_ 10 nm), and the Ti membrane with a 100 nm TiO_2_ layer deposited by ALD (Ti-TiO_2_ 100 nm) were analyzed using SEM and 3D surface texture measurements. Figure 2a illustrates the regular pyramidal surface structure on a large scale, confirming a uniform morphological transformation across the 1 × 1 cm^2^ Ti foil. As seen in Figure 2b, the pyramids feature square bases about 85 μm wide and show visible brittleness at the top regions. The entire pyramidal surface contains rough artifacts resulting from laser ablation. Figure S1 presents a cross-sectional view of the pyramids, showing rough walls and signs of damage at the tops. The 3D surface analysis further confirmed that the structured area maintains a uniform and consistent surface profile. Combined with SEM cross-sectional analysis, these measurements revealed a height range of 300–400 μm from the base of the foil to the apex of the pyramids. A detailed ISO 25178 surface texture analysis is presented in Table S1. The shapes of pyramidal structures are defined by scanning regime applied to the entire surface. The scanning regime is based on a mesh strategy with a tight spot overlap (83%), resulting in significant energy confinement in a subsurface region. The choice of the laser fluence of 1.3 J/cm^2^, which is significantly above the typical ablation threshold for titanium, reported to be around 0.2–0.3 J/cm^2^, combined with a high repetition rate (1 MHz) and dense spot overlap (83%), ensures highly efficient ablation.^31^ Furthermore, the brittle morphology observed at the apex of the pyramids (Figures 2b and S1) might be related to the formation of titanium oxides during laser processing. The associated thermal gradients and rapid solidification may induce residual stresses, making the oxidized regions susceptible to fracture. This hypothesis is supported by XPS analysis, which confirms the presence on surface TiO_2_. The inherently brittle nature of the oxide layer compared to metallic Ti likely contributes to the observed structural damage.

**Figure 2:**
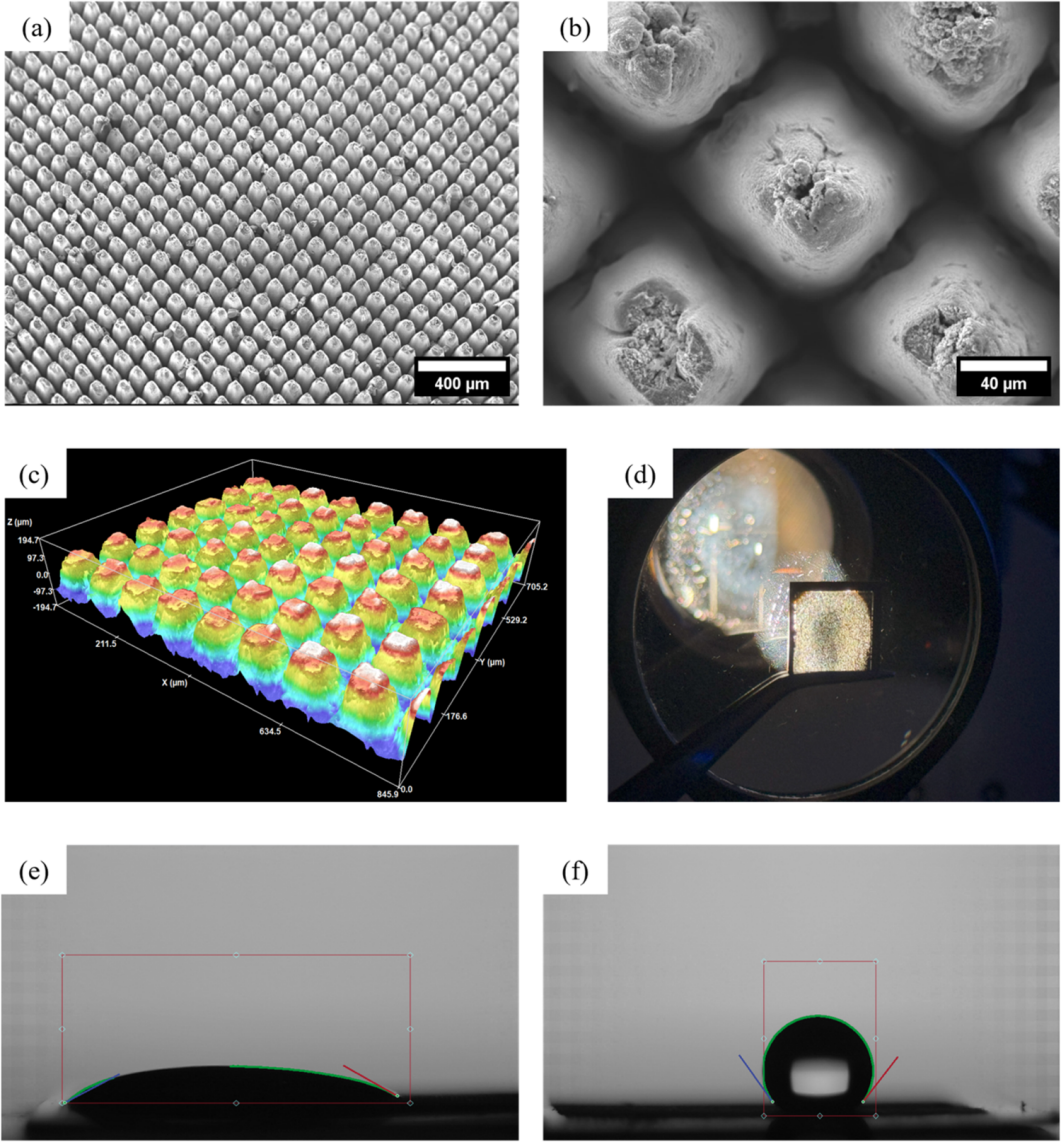
(a) Pyramidal surface structure on the Ti membrane, (b) magnified view of the pyramidal surface, (c) the 3D surface texture scan of the Ti membrane, (d) the light transmission test, (e) the contact angle measurement of the Ti membrane, and (f) the contact angle measurement after ALD deposition of TiO_2_.

Additionally, cross-sectional SEM images confirmed that the laser had drilled entirely through the Ti foil. This was further verified by direct irradiation using a 300 W Xe lamp (Figure 1d), where light transmission through the drilled holes further supports the classification of the material as a Ti membrane after modification. Contact angle measurements demonstrated that after laser modification, the surface became highly hydrophilic, whereas ALD deposition rendered the surface hydrophobic, which is a characteristic behavior of TiO_2_ ALD layers.^32^ This behavior is attributed to the low density of surface hydroxyl groups and residual chlorine species resulting from the TiCl_4_/H_2_O ALD process, both of which reduce surface polarity and limit water adsorption.^33^ Detailed contact angle measurement results are provided in Table S2.

### 3.2 Chemical and Optical Properties

Raman spectroscopy was conducted to analyze the chemical composition of the developed composites. Figure 3a presents the Raman spectra of the Ti foil, Ti membrane, Ti-TiO_2_ (10 nm), and Ti-TiO_2_ (100 nm) samples. As a result of laser ablation, not only did the surface morphology change, but the surface also oxidized, as indicated by the formation of TiO_2_ phases. This transformation is expected to represent one of the standard methods for TiO_2_ formation.^34^ The observed peak at 152 cm^–1^ corresponds to the Eg mode of anatase TiO_2_, but the shift from the typical position could be due to structural defects or phase mixing effects.^35^ The detection of Raman bands at around 440 and 610 cm^–1^, corresponding to the Eg and Ag modes of rutile, further supports the presence of mixed TiO_2_ phases.^36^ After the deposition of a 10 nm TiO_2_ layer by ALD, shifts were observed in all major peaks. Specifically, the peaks shifted to 146, 440, and 605 cm^–1^, which can be explained by several factors. The shift to 146 cm^–1^ may be attributed to oxygen deficiency, phonon confinement, or extrinsic doping, reflecting the sensitivity of this peak to structural and compositional changes.^37^ The shifts at 440 and 605 cm^–1^ may be due to an amorphous layer formed during the thin ALD coating and its influence on local structural disorder.^38,39^ With the deposition of a 100 nm TiO_2_ layer, a crystalline phase emerges.^40^ This is confirmed by the sharpening of the Raman peaks and their appearance at 144, 395, 515, and 633 cm^–1^, which correspond to the characteristic modes of crystalline anatase TiO_2_, as widely reported in the literature.^41,42^ Figure 3d presents a Raman mapping image of the 144 cm^–1^ peak, confirming homogeneous surface coverage of the Ti membrane by the 100 nm ALD layer.

**Figure 3:**
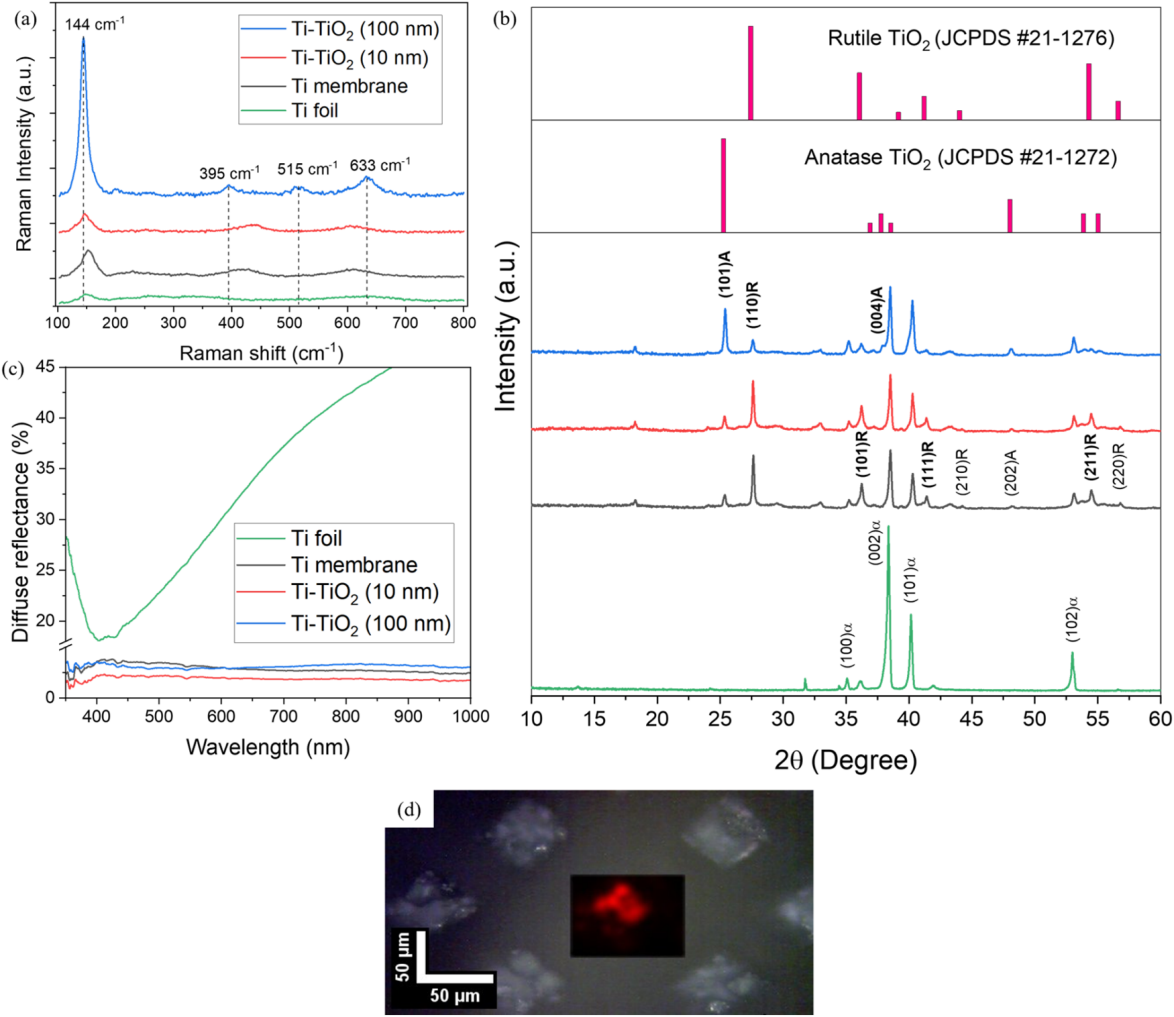
(a) Raman spectra of Ti foil, Ti membrane, Ti-TiO_2_ (10 nm), and Ti-TiO_2_ (100 nm) samples; (b) XRD patterns of the same samples, with reference diffraction patterns of anatase (JCPDS 21-1272) and rutile (JCPDS 21-1276) inserted for phase identification. Dominant peaks are annotated to highlight crystallinity trends induced by ALD; (c) diffuse reflectance spectra; and (d) Raman mapping of the Ti-TiO_2_ (100 nm) sample, highlighting the 144 cm^–1^ peak in red.

To support the Raman findings, XRD analysis (Figure 3b) was conducted to investigate the long-range crystallographic structure of the synthesized materials. The untreated Ti foil exhibited characteristic reflections of hexagonal α-Ti at 2θ = 35.1° ((100)), 38.4° ((002)), 40.2° ((101)), and 53.0° ((102)), consistent with reference data.^43^ Additional weak peaks were attributed to surface oxidation or impurities. The XRD pattern of the laser-processed Ti membrane revealed the formation of mixed-phase TiO_2_ corresponding to both anatase and rutile phases. The anatase phase indicates peaks at 2θ = 25.4° ((101)) and 48.3° ((202)), while the rutile phase showed peaks at 27.6° ((110)), 36.2° ((101)), 41.4° ((111)), 44.2° ((210)), 54.5° ((211)), and 56.8° ((220)). These peak positions correspond with values reported in the literature.^44^

The formation of a TiO_2_ layer led to a decrease in Ti foil peak intensities without detectable shifts in their positions, which indicates partial surface coverage without significant substrate lattice distortion. Subsequent ALD (10 nm TiO_2_ layer) did not result in new diffraction peaks or noticeable intensity changes, confirming the amorphous nature of the thin ALD film. In contrast, the 100 nm ALD layer induced enhanced diffraction features. Specifically, the intensity of anatase reflections at 25.4° ((101)) and 48.3° ((202)) increased markedly, and a new anatase (004) peak appeared at 37.9°, indicating crystallization and preferential orientation of the TiO_2_ layer. Simultaneously, a reduction in rutile peak intensities was observed, suggesting a phase shift toward a predominantly anatase structure with increased ALD thickness.

These complementary analyses confirm the formation of mixed-phase TiO_2_, with both anatase and rutile phases present. The anatase phase becomes predominant after deposition of the 100 nm ALD TiO_2_ layer. The improved crystallinity and phase composition are expected to influence the photoelectrochemical behavior of the fabricated membranes.

The optical properties of the samples were characterized through diffuse reflectance analysis. The Ti foil showed high reflectivity in the visible and near-infrared (NIR) regions, but its diffuse reflectance reached over 40% (>900 nm). In contrast, the laser-processed Ti membrane showed a significant reduction in reflectance, with values dropping below 5% across the entire spectrum. This decrease is attributed to the microstructuring of the Ti surface and the formation of a thin amorphous TiO_2_ layer. As depicted in Figure 1c, the laser-processed areas exhibit a significant darkening effect, characterized by a deep black coloration. Since no significant improvement in light absorption was observed after depositing 10 and 100 nm TiO_2_ layers via ALD, it can be concluded that the laser-induced surface structure plays the dominant role in boosting broadband absorption. The results clearly indicate that combining microstructured surfaces with TiO_2_ coatings enhances light absorption across the solar spectrum, which is crucial for improving PEC water splitting efficiency. Due to the highly structured nature of the Ti-TiO_2_ membranes, accurate optical bandgap determination using Tauc plot analysis could not be reliably performed, as scattering and surface morphology effects significantly distorted the absorption edge in diffuse reflectance measurements.

### 3.3 XPS Analysis

XPS analysis was conducted to investigate the chemical state evolution of fabricated samples. The survey spectra reveal mainly titanium (Ti), oxygen (O) and carbon (C) for all samples (Figure S2). In the case of pristine Ti foil, one may observe additional impurities like, calcium (Ca), silicon (Si), sulfur (S) and nitrogen (N).

The core-level spectra for pristine Ti foil, laser-nanostructured Ti surface, and ALD-modified samples reveal distinct transformations in surface composition and oxidation state (Figure 4). In the case of the untreated Ti foil, the Ti 2p_3/2_ and Ti 2p_1/2_ peaks are observed at binding energies of approximately 454.0 and 460.5 eV, respectively, with a spin–orbit splitting of ∼6.5 eV. The observed peak positions correspond to metallic Ti (Ti^0^), suggesting that the surface remains unoxidized mainly. The sharp and symmetric shape of the peaks indicates a clean, well-structured surface. However, a minor shoulder near 458.5 eV may point to the presence of a thin native oxide layer, such as TiO_2_ or suboxides like Ti_2_O_3_, which typically develop upon air exposure.^45^ Following laser treatment, the Ti 2p spectrum experiences a notable alteration, characterized by the significant peaks migrating to elevated binding energies: Ti 2p_3/2_ around 458.5 eV and Ti 2p_1/2_ close to 464.3 eV. This shift suggests the presence of the Ti4^+^ oxidation state linked to TiO_2_. The metallic Ti signal is either absent or significantly suppressed, suggesting extensive surface oxidation induced by the high-power laser pulses. The broadened and asymmetric peaks indicate a mixture of titanium oxidation states, including Ti^3+^ and Ti^2+^, which likely result from the complex thermal and oxidation processes triggered by femtosecond laser irradiation in air.^45,46^ Following the ALD deposition of a 10 nm TiO_2_ layer, the Ti 2p core-level spectrum is dominated by sharp and symmetric peaks at 458.8 eV (Ti 2p_3/2_) and 464.6 eV (Ti 2p_1/2_), corresponding to stoichiometric Ti^4+^ in TiO_2_. No detectable signals from metallic Ti or suboxide species are present, confirming the formation of a uniform and fully oxidized TiO_2_ overlayer. The reduced peak width and increased intensity reflect the high chemical purity, uniformity, and conformality of the ALD coating.

**Figure 4:**
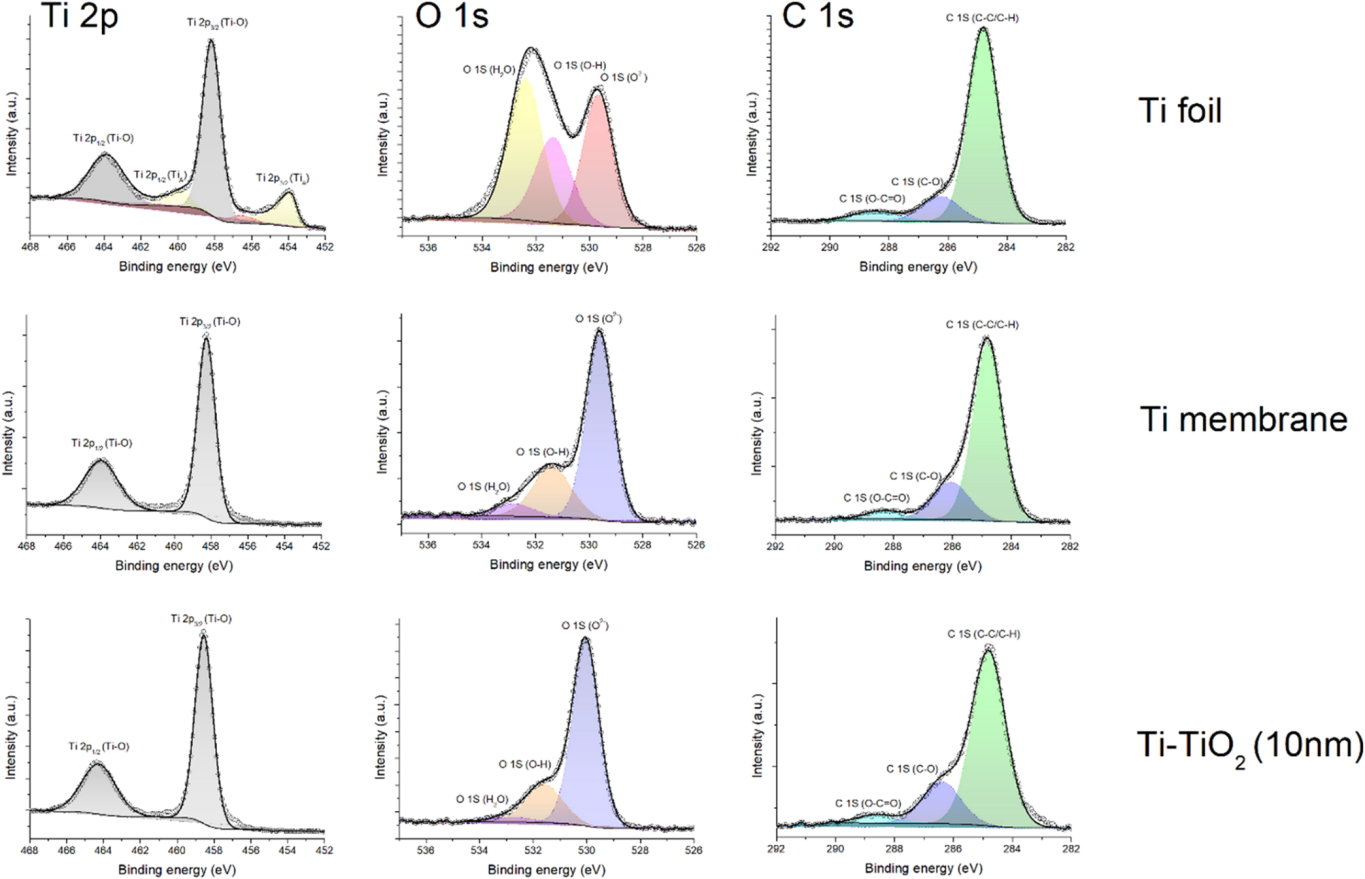
Core-level spectra of pristine Ti foil, laser-nanostructured Ti surface, and ALD-modified samples.

A comparative analysis of the evaluation of the O 1s spectra was conducted as well. The O 1s spectrum for Ti foil displays a broad asymmetric peak centered around 530.0 eV, which corresponds to lattice oxygen (O^2–^) in stoichiometric TiO_2_. This observation suggests the presence of a thin native oxide layer that forms spontaneously on titanium when exposed to ambient conditions. A minor shoulder near 531.5 eV is also observed, which is attributed to surface hydroxyl groups (−OH) or adsorbed water molecules commonly associated with environmental exposure to the native oxide surface. The O 1s spectral envelope becomes significantly more intense and asymmetric upon high-power femtosecond laser processing. The main peak remains at ∼ 530.0 eV, consistent with TiO_2_. A more pronounced shoulder appears in the 532.0–532.5 eV region, indicating a higher concentration of surface hydroxyl groups, adsorbed water molecules, and possibly substoichiometric titanium oxides (TiO_2–*x*_).^29^ This effect is likely due to more surface area, more defects, and greater oxygen content on the surface. The O 1s spectrum of the ALD modified sample exhibits a distinctly sharper and more intense peak centered at 530.0 eV, accompanied by a notable reduction in asymmetry. The limited presence of the higher-binding energy component indicates a dense, conformal, and stoichiometric TiO_2_ coating characterized by minimal hydroxylation and surface impurities. The narrow fwhm and prominent lattice oxygen peak validate the exceptional chemical purity and uniformity of the ALD-grown TiO_2_ film, which efficiently passivates the underlying laser-structured surface.

Finally, the high-resolution C 1s XPS spectra revealed distinct chemical states of carbon across the three samples. For pristine titanium, the C 1s spectrum is mainly centered at ∼284.8 eV, consistent with surface contamination from C–C/C–H species, and includes minor peaks from oxygenated carbon compounds like C–O and O–C=O. After femtosecond laser processing, a noticeable rise in carbon intensity was observed, along with the appearance of a new feature at ∼282.0 eV, indicating the formation of Ti–C bonds likely caused by laser-driven surface modifications. In contrast, the ALD-coated Ti showed a significant reduction in carbon contamination and a sharper Ti–C component, suggesting interfacial carbide stabilization by the ALD process.^47^

We also employed the valence band X-ray photoelectron spectroscopy (VB-XPS) to evaluate the evolution of surface electronic structure (Figure S3). These measurements and their analysis provide insights into the modification of the valence density of states (DOS) resulting from laser surface processing and TiO_2_ thin-film deposition.

The untreated Ti foil shows a broad and strong valence band spectrum, with a leading edge starting around ∼2.6–2.7 eV from the Fermi level. The elevated density of states ranging from approximately 3 eV to beyond 10 eV aligns with the metallic characteristics of Ti, primarily influenced by the hybridization of Ti 3d and 3p states. The pronounced valence band maximum (VBM) and elevated intensity are indicative of bulk metallic Ti, reinforcing the notion that there is no substantial oxide layer present at the surface.

The Ti membrane exhibits a notable change in the valence region. The spectrum indicates a shallower VBM around 2.1–2.2 eV, with a noteworthy redistribution of spectral intensity toward midbinding energies. The observed features indicate the development of a surface oxide resembling TiO_2_, potentially consisting of both amorphous and crystalline phases, whether formed naturally or through laser induction.^45^

The sample modified by ALD exhibits a VBM around 2.3–2.5 eV, along with a broadened valence band that extends up to approximately 10 eV. The spectrum exhibits features typical of stoichiometric TiO2, highlighting significant contributions from O 2p nonbonding states and Ti 3d-O 2p hybridized states, aligning with an anatase-like electronic structure.^45^ The lack of notable sub-bandgap characteristics close to the Fermi level implies a minimal presence of Ti^3+^ or oxygen vacancy states, pointing to a well-structured oxide layer. The spectrum shows a more advanced and organized valence band, indicating the successful creation of an ultrathin TiO_2_ coating and the reduction of contributions from the metallic Ti surface, which aligns closely with the XPS core-level analysis.

### 3.4 Photoelectrochemical Performance

The PEC behavior of the Ti-based electrodes was first evaluated using electrochemical methods, including CV, CA, and LSV, performed under both dark and light conditions. CV tests were conducted across a range of scan rates (20–500 mV/s). Representative CV curves at 50 mV/s are shown in Figure 5a, while the complete data set is provided in Figure S4. The Ti membrane exhibited a moderate photocurrent response with clear scan rate dependence, suggesting a predominantly capacitive behavior likely associated with native oxide formation and surface roughness introduced by laser processing. The photocurrent for the Ti-TiO_2_ (10 nm) sample was lower, likely because the amorphous ALD coating limited its ability to absorb light efficiently and facilitate charge transfer. Moreover, the hydrophobic surface promotes oxygen bubble entrapment at the electrode–electrolyte interface, reducing the effective contact area and increasing charge-transfer resistance. This interfacial effect becomes particularly critical for ultrathin TiO_2_ films, where limited wettability further impairs PEC efficiency.^48^ In contrast, the Ti-TiO_2_ (100 nm) sample exhibited the highest photocurrent, accompanied by a more significant variation with scan rate. The findings indicate enhanced charge storage at the surface and increased light absorption, likely due to the thicker and more crystalline TiO_2_ film. To better understand how charge is stored, peak current was plotted versus scan rate and log (scan rate), as seen in Figure S5a,b. The linear trend in the peak current density versus scan rate plot suggests predominantly capacitive behavior across all samples, with steeper slopes indicating a larger surface-controlled contribution. For a more detailed understanding, b-values were extracted from the log–log plots according to the following relationship:^49^

3 $$\log\left(\mathrm{peak current density}\left(\frac{mA}{{cm}^{2}}\right)\right)=b\times \log\left(scan\;rate\left(\frac{mV}{s}\right)\right)+\log\left(a\right)$$

**Figure 5:**
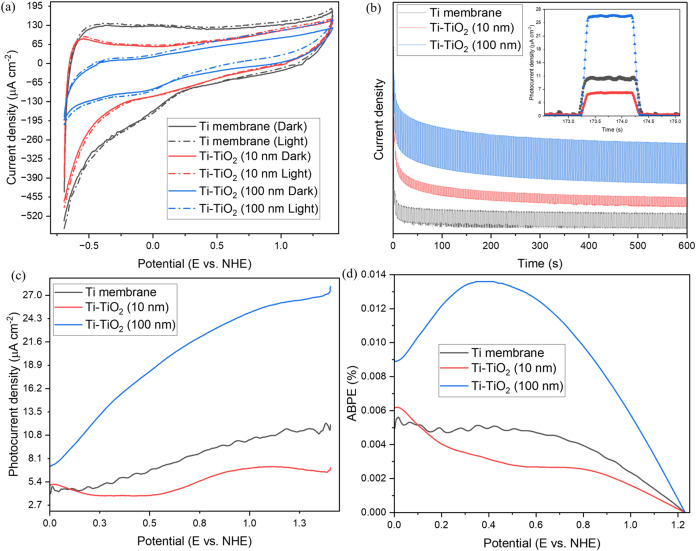
(a) Cyclic voltammetry measurements of all samples at a scan rate of 50 mV/s, (b) chronoamperometry analysis with an inset highlighting one light ON/OFF cycle, (c) photocurrent density from linear sweep voltammetry (LSV) measurements, and (d) calculated applied bias photon-to-current efficiency (ABPE).

The Ti-TiO_2_ (100 nm) sample showed the highest photocurrent along with sharp and stable transitions during the light ON/OFF cycles, as illustrated in the inset of Figure 5b. This points to a fast photoresponse and good photostability for all the samples. In addition, 3-h chronoamperometry ON/OFF photostability measurements were performed to evaluate the operational stability of the Ti-TiO_2_ membranes. The corresponding data are provided in the Supporting Information (Figure S6). LSV measurements were carried out to further assess oxidation activity (Figure 5c). The Ti-TiO_2_ (100 nm) electrode exhibited a steady increase in photocurrent across the entire potential range, reaching around 27 μA/cm^2^ at 1.4 V vs NHE. A photocurrent of ∼11 μA/cm^2^ was recorded for the Ti membrane, with the 10 nm TiO_2_-coated sample exhibiting reduced efficiency. This supports the idea that the thin ALD layer may act more as a barrier to charge transport than as an active photoabsorber. The results of LSV measurements under illumination and in the dark are presented in Figure S7. To quantify PEC performance, ABPE was also calculated and is presented in Figure 5d. The 100 nm-coated sample yielded the optimal result, attaining a peak ABPE of 0.014% at 0.39 V vs NHE, about thrice greater than both the untreated and 10 nm-coated electrodes. The diminished ABPE values in the thinner and uncoated samples indicate inferior charge separation and probable recombination losses.

It is worth highlighting that although Liang et al.^27^ reported higher photocurrent densities for TiO_2–*x*_ photoelectrodes, their enhancement primarily originated from oxygen vacancy introduction, which may negatively affect long-term operation due to enhanced recombination and chemical instability under continuous illumination. In contrast, our ALD-based approach provides highly conformal, stoichiometric TiO_2_ coatings with controlled crystallinity and superior adhesion to the laser-structured substrate. The combination of hierarchical structuring and defect-free crystalline coatings results in stable photoelectrode operation, ensuring mechanical integrity and sustained PEC performance without deterioration over extended periods.

Electrochemical impedance spectroscopy (EIS) and Mott–Schottky (MS) analyses were conducted under dark and illuminated conditions to investigate the interfacial charge transport characteristics and semiconducting behavior of the fabricated electrodes. The Nyquist plots (Figure 6a) were fitted using a Randles-type equivalent circuit, commonly applied to TiO_2_ ALD interfaces.^50,51^ This model includes the solution resistance (Rs), charge transfer resistance (Rct), and a constant phase element (CPE) to account for nonideal capacitive behavior at the electrode–electrolyte interface. The extracted fitting parameters are summarized in Table S3. The Ti membrane exhibited high Rct values of 76.17 kΩ (dark) and 73.99 kΩ (light), indicating sluggish charge transfer kinetics. The deposition of a 10 nm TiO_2_ layer by ALD significantly reduced the Rct values to 47.69 kΩ (dark) and 52.79 kΩ (light). Notably, the slightly elevated Rct observed under light suggests that the TiO_2_ layer could introduce trap states or facilitate surface recombination, which may interfere with efficient charge transport.^52–54^ The Ti-TiO_2_ (100 nm) electrode exhibited the lowest Rct values, with 40.53 kΩ (dark) and 39.90 kΩ (light), indicating substantially improved charge transfer characteristics due to the increased thickness and crystallinity of the TiO_2_ layer. It is also noteworthy that the solution resistance (Rs) for the 10 nm-coated sample was slightly elevated (16.66 Ω dark, 16.09 Ω light) compared to the Ti membrane (∼14.7 Ω), likely due to interfacial resistance introduced by the amorphous ALD film. In contrast, Rs decreased significantly in the 100 nm-coated electrode, reaching 3.41 Ω under illumination, reflecting improved electronic contact and lower electrolyte resistance. A drop in the CPE exponent (α) from 0.91 (Ti membrane) to 0.71 (Ti-TiO_2_ 100 nm, under illumination) points to greater surface irregularity, likely caused by the formation of the nanostructured TiO_2_ coating. To further analyze the semiconductor properties, Mott–Schottky (MS) plots were generated (Figure 6b) to determine the flat-band potential (V_fb_) and acceptor concentration (N_a_) of the electrodes, assuming p-type semiconductor behavior. These values were extracted by linear fitting of 1/C_sc_^2^ versus applied potential, using the following equation:^55^

4 $$\frac{1}{C^{2}}=\frac{2}{\varepsilon \varepsilon_{0}qN_{a}A^{2}}\left(E-E_{fb}-\frac{k_{B}T}{q}\right)$$

where *C* is the capacitance of the space-charge layer, ε is the dielectric constant of TiO_2_, ε_0_ is the vacuum permittivity, *q* is the elementary charge, *N*_a_ is the acceptor density, *A* is the electrode area, *E* is the applied potential, *E*_fb_ is the flat-band potential, *k*_B_ is the Boltzmann constant, and *T* is the absolute temperature.

**Figure 6:**
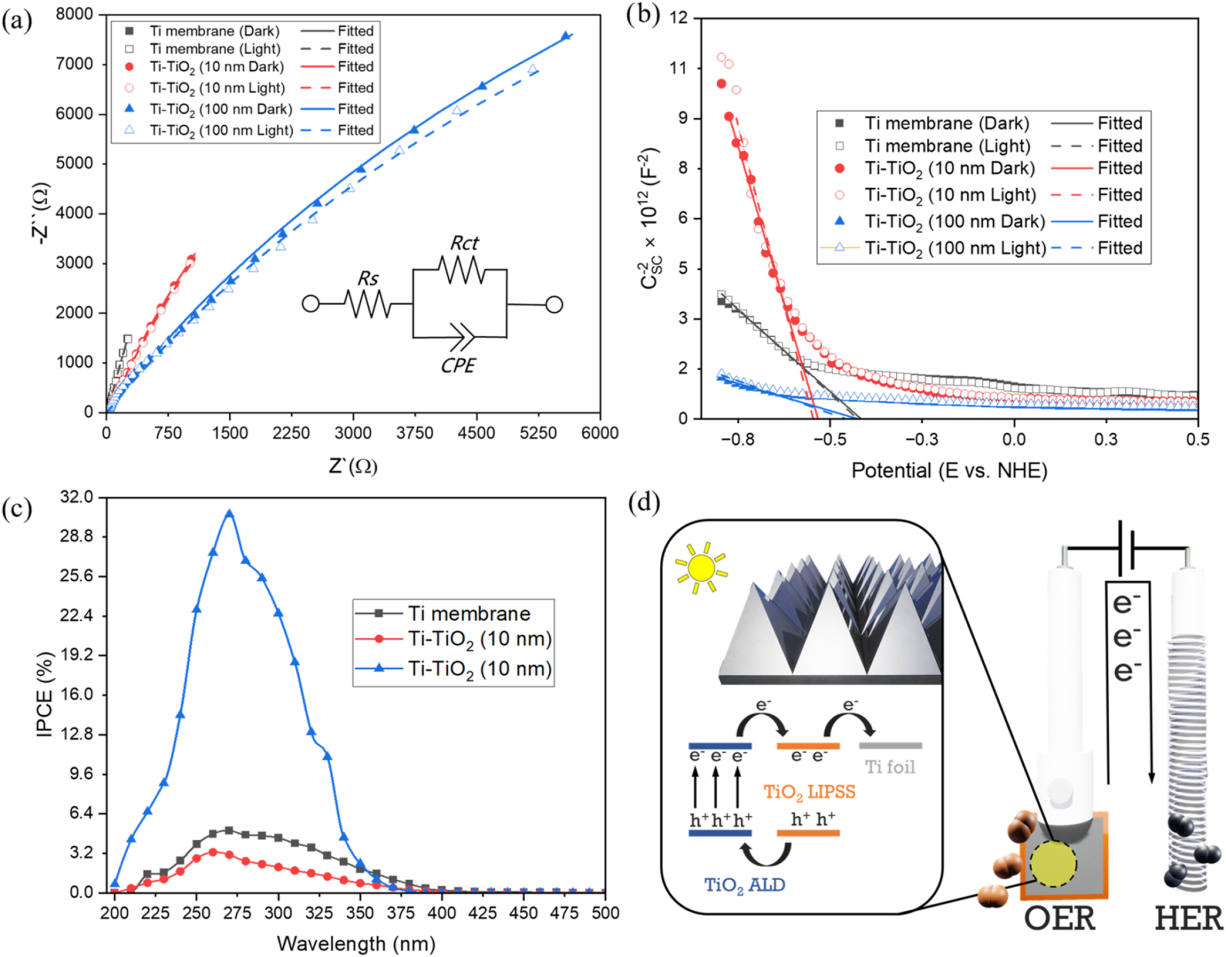
(a) Electrochemical impedance spectroscopy data for all samples, (b) Mott–Schottky plots, (c) incident photon-to-current efficiency measurements, and (d) schematic illustration of the photoelectrochemical behavior and charge transfer mechanism.

The extracted data are summarized in Table S4. The Ti membrane exhibited flat-band potentials of −0.44 V (dark) and −0.45 V (light) vs NHE, with acceptor concentrations in the range of (1.39–1.44) × 10^19^ m^–3^. The 10 nm TiO_2_-coated sample displayed more negative flat-band potentials (−0.56 V dark, −0.57 V light) and significantly lower acceptor concentrations (3.28–3.76 × 10^18^ m^–3^), suggesting reduced carrier availability and a suppressed charge transport capability. These results are consistent with the lower photocurrent and ABPE values observed in PEC tests, likely due to the amorphous nature and higher defect density in the ultrathin film. Compared to other samples, the 100 nm TiO_2_-coated electrode showed flat-band potentials between −0.45 and −0.48 V and a much higher acceptor concentration (∼4.34 × 10^19^ m^–3^), pointing to improved p-type conductivity and better overall electronic behavior. The p-type behavior in all electrodes is likely influenced by surface and interfacial defect states,^56^ especially in the Ti-TiO_2_ (10 nm) sample, where these effects appear to dominate the overall electronic response. While TiO_2_ is normally an n-type semiconductor, apparent p-type behavior in Mott–Schottky plots has been reported in thin films exhibiting high concentrations of surface defects or nonstoichiometric phases.^56,57^ In our case, the oxygen vacancies and Ti^3+^ defect states evidenced by XPS and VB-XPS, combined with the mixed anatase/rutile phases identified by XRD, likely result in band bending or surface inversion effects that explain the positive slope without indicating true bulk p-type conductivity. The observed trend toward improved conductivity and lower interfacial resistance with increasing TiO_2_ thickness confirms that a crystalline, nanostructured TiO_2_ layer can overcome charge transfer limitations imposed by defects from laser structuring and thin amorphous films. Together, the EIS and Mott–Schottky results reinforce PEC findings by highlighting that the 100 nm TiO_2_ layer provides faster charge transfer, lower solution and interfacial resistance, higher acceptor concentration, and ultimately, enhanced photoelectrochemical performance.

IPCE measurements were conducted to evaluate the wavelength-dependent photoactivity of the fabricated electrodes, as shown in Figure 6c. The IPCE values were calculated using the equation:^58^

5 $$\mathrm{IPCE}\left(\lambda \right)=1240\times \frac{J_{\mathrm{sc}}}{P_{\mathrm{in}}\left(\lambda \right)\times \lambda }$$

where λ is the wavelength in nanometers (nm), *J*_sc_ is the measured photocurrent density (mA/cm^2^), and *P*_in_(λ) is the incident light power density at the corresponding wavelength (mW/cm^2^).

The measurements were performed using a monochromator to isolate specific wavelengths. At each selected wavelength, the incident light intensity was recorded with a calibrated photodetector, and the photocurrent response of the electrodes was measured. All samples showed a clear photoresponse in the UV region (200–370 nm), which aligns with the wide band gap of TiO_2_. Among the tested electrodes, the Ti-TiO_2_ (100 nm) sample achieved the highest IPCE, reaching around 31% at 275 nm. This suggests better light absorption and more effective charge carrier separation. The Ti membrane showed a moderate IPCE response (∼6.5%), likely due to its laser-induced surface texturing and the formation of mixed-phase TiO_2_ during processing. In comparison, the Ti-TiO_2_ (10 nm) sample had the lowest IPCE (∼3.5%), confirming the limited performance of the thin, amorphous TiO_2_ layer. These results align with photocurrent, ABPE, and EIS data, underscoring the significance of TiO_2_ thickness and crystallinity in enhancing the PEC behavior of Ti-based electrodes.

To summarize and better explain the photoelectrochemical behavior and charge transfer processes of the prepared electrodes, a conceptual schematic is shown in Figure 6d. In this diagram, illumination triggers the generation of electron–hole pairs within the TiO_2_ regions, both from the laser-induced oxide layer and the ALD-deposited TiO_2_ coating. The photogenerated holes move toward the electrode–electrolyte interface, driving the oxygen evolution reaction (OER). At the same time, the electrons are transported through the conductive Ti base and collected at the counter electrode to carry out the hydrogen evolution reaction (HER). This configuration facilitates efficient charge separation, driven by the synergy between the laser-structured surface and the conformal TiO_2_ layer, which together enhance light absorption and optimize charge transport pathways. Based on the electrochemical and spectroscopic results, the 100 nm ALD TiO_2_ layer enhances carrier generation and transfer. This is supported by the observed increase in photocurrent, lower charge transfer resistance, and higher incident photon-to-current efficiency (IPCE). In addition, the laser-structured Ti membrane significantly contributes to light harvesting, enhancing the PEC performance of both coated and uncoated electrodes by increasing the surface area and promoting effective photon capture. Post-PEC SEM and XRD measurements were performed to evaluate changes in surface morphology and crystal structure after photoelectrochemical tests. No significant changes were observed, confirming that the pyramidal morphology and phase composition remained stable under the applied PEC conditions. The data are presented in the Supporting Information (Figure S8).

To assess our UV PEC performance against recent TiO_2_ systems, we note that hierarchical photonic-crystal TiO_2_ nanotubes achieved photocurrent densities of approximately 1.4 mA/cm^2^ at around 1.23 V versus NHE under UV light,^59^ significantly higher than our 27 μA/cm^2^. However, this performance relied on complex templated architectures and multilayer photonic structures that involve precise design and fabrication steps. In comparison, Au-decorated TiO_2_ nanotubes produced a photocurrent of only approximately 15.8 μA/cm^2^ under UV illumination, despite the use of noble metal enhancement.^60^ Our binder-free and freestanding Ti-TiO_2_ membrane reaches ∼27 μA/cm^2^ and ∼31% IPCE at 275 nm, without the need for noble metals, transparent substrates, or templated morphology. This demonstrates a practical and structurally robust UV photoelectrode platform that can serve as a reproducible and scalable baseline for further development.

We can conclude that the enhanced PEC performance observed in our system is attributed to the synergistic effects of hierarchical structuring, controlled composition, which is reached by ALD, and modified optical properties. The femtosecond laser-induced microstructures significantly increase light harvesting by enhancing multiple internal reflections and scattering, as supported by the UV–vis absorption spectra. The conformal ALD-deposited TiO_2_ layer provides high crystallinity and uniform coverage of the complex 3D morphology, ensuring efficient charge separation and suppressing bulk recombination. Additionally, laser-induced oxygen vacancies and Ti^3+^ states, identified by XPS and VB-XPS, introduce shallow trap states that may facilitate sub-bandgap absorption and enhance charge carrier dynamics at the semiconductor-electrolyte interface. The coexistence of anatase and rutile phases, as revealed by XRD, may further promote interfacial charge separation due to favorable band alignment. The structural and compositional properties collectively boost photocurrent density, lower onset potential, and improve PEC activity as evidenced by our data.

## 4 Conclusions

This study presents an innovative fabrication method for TiO_2_-based photoanodes that combines femtosecond laser-induced hierarchical structures with ALD. Laser processing generates microstructured surfaces that improve light trapping and augment the active surface area. Simultaneously, ALD facilitates the deposition of conformal, highly crystalline TiO_2_ coatings with precise thickness control and superior interfacial adhesion. This binder-free connection guarantees mechanical durability and enhanced stability during PEC operation. While the absolute photocurrent density achieved in this study is inferior to that of leading TiO_2_ photoanodes, our principal aim was to develop a highly controllable and scalable manufacturing platform to produce stable and structurally designed photoelectrodes. The integrated laser-ALD technique has considerable potential for optimization through compositional adjustment, cocatalyst incorporation, and heterostructure development, thereby improving PEC performance. This adaptable platform has the potential to provide a robust foundation for the future advancement of durable and efficient PEC systems for solar water splitting and associated applications.

## Supplementary Material



## Notes

Cross-sectional view of the pyramidal structures on the Ti membrane; surface roughness of the pyramid walls; damaged and intact pyramid tops of the Ti membrane; SEM image and XRD analysis of the Ti-TiO_2_ (100 nm) sample after PEC tests; 3D surface texture measurement results of the Ti membrane according to the ISO 25178 standard; contact angle measurements of Ti membrane, Ti-TiO_2_ (10 nm), and Ti-TiO_2_ (100 nm) samples; XPS survey spectra of pristine Ti foil, laser-nanostructured Ti surface, and ALD-modified samples; valence band XPS spectra of pristine Ti foil, laser-nanostructured Ti surface, and ALD-modified samples; cyclic voltammetry measurements at various scan rates for Ti membrane under dark and illuminated conditions, Ti-TiO_2_ (10 nm) under dark and illuminated conditions, Ti-TiO_2_ (100 nm) under dark and illuminated conditions; Linear sweep voltammetry voltammetry measurements of all samples at a scan rate of 50 mV/s; peak current density versus scan rate and log­(peak current density) versus log­(scan rate) for all prepared samples; fitted EIS parameters for the measured samples; fitted Mott–Schottky parameters for the measured samples; chronoamperometric measurement conducted over a period of 3 h for the Ti-TiO_2_ (100 nm) sample (PDF)
