# Supplementary material for: Hierarchically Structured Ti-TiO2 Membranes Fabricated by Femtosecond Laser Ablation and Atomic Layer Deposition for Enhanced Photoelectrochemical Water Splitting
Source: ACS Appl Mater Interfaces. 2025 Jul 21;17(30):43390–402. doi: 10.1021/acsami.5c07488 (PMC12314867; doi:10.1021/acsami.5c07488)
Supplement: Supplementary file 1 [file am5c07488_si_001.pdf]

Supporting information

Hierarchically Structured Ti-TiO_2 Membranes Fabricated by Femtosecond Laser Ablation and Atomic Layer Deposition for Enhanced Photoelectrochemical Water Splitting

Andrii Lys[†], *Iaroslav Gnilitskyi*[‡§], *Emerson Coy*[†], *Mariusz Jancelewicz*[†], *Mikhael Bechelany*[¡¿], *Igor Iatsunskyi*[*†]

[†]NanoBioMedical Centre, Adam Mickiewicz University, 3, Wszechnicy Piastowskiej Str., 61-614 Poznan, Poland, *email: igoyat@amu.edu.pl

[‡]“NoviNano” Lab LLC, Pasternaka, 5, 79015 Lviv, Ukraine

[§]Department of Applied Physics and Nanomaterials Science, Lviv Polytechnic National University, 12, Bandery Str., 79013 Lviv, Ukraine

[¡]Institut Européen des Membranes, IEM, UMR 5635, University of Montpellier, ENSCM, CNRS, 34090 Montpellier, France.

¿Functional Materials Group, Gulf University for Science and Technology (GUST), Mubarak Al-Abdullah 32093, Kuwait

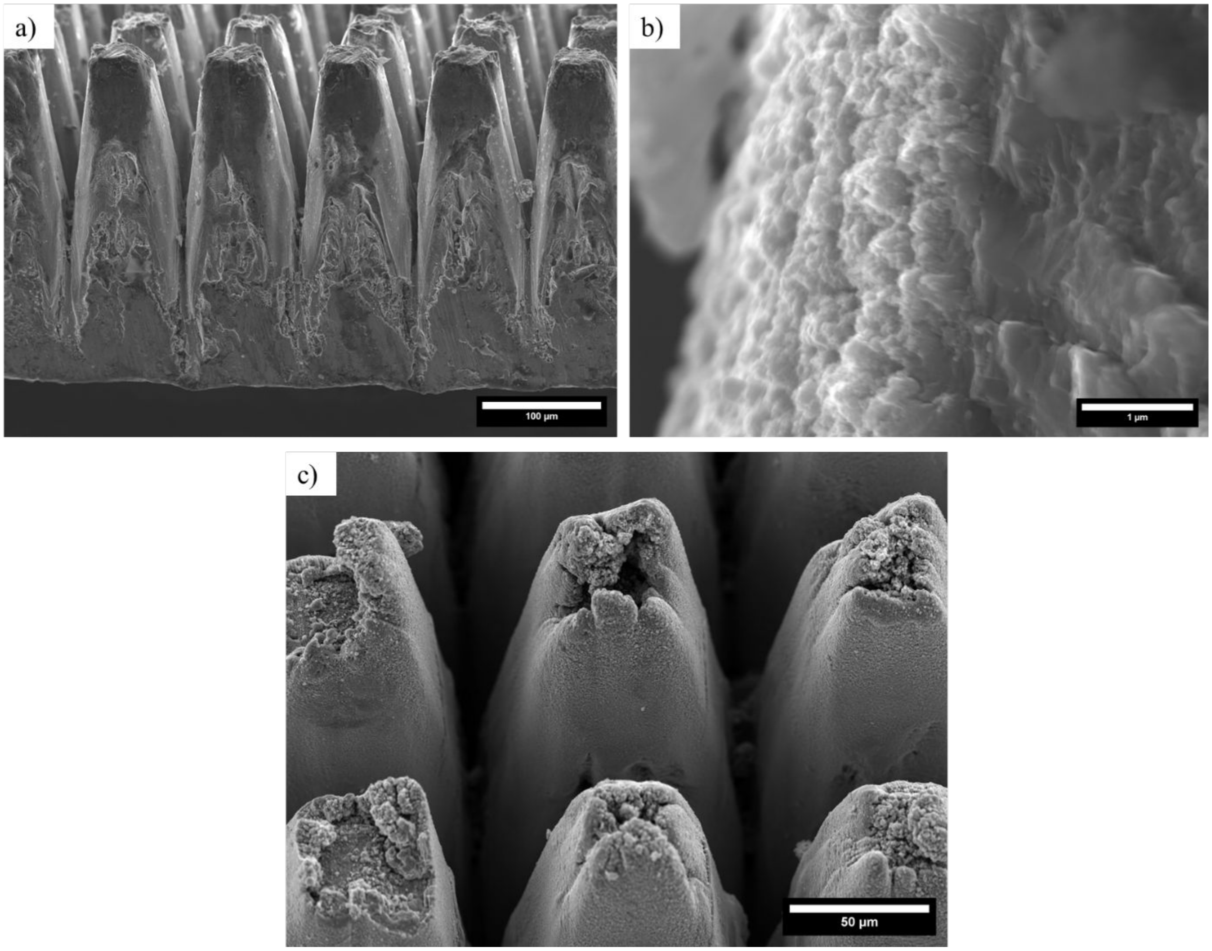

Figure S1. (a) Cross-sectional view of the pyramidal structures on the Ti membrane, (b) surface roughness of the pyramid walls, and (c) damaged and intact pyramid tops of the Ti membrane.

Table S1. 3D surface texture measurement results of the Ti membrane according to the ISO 25178 standard.

Sa (Arithmetic Mean Height)	42.799 µm	Sq (Root Mean Square Height)	52.274 µm
Sku (Kurtosis)	2.4580	Ssk (Skewness)	0.3273
Smean (Mean Surface Height)	-28.230 µm	Sv (Maximum Pit Depth)	179.55 µm
Sp (Maximum Peak Height)	213.90 µm	Sz (Maximum Height)	393.45 µm

Table S2. Contact angle measurements of Ti membrane, Ti-TiO_2 (10 nm), and Ti-TiO_2 (100 nm) samples.

**Sample name**	**Left Angle (Â°)**	**Right Angle (Â°)**	**Average Angle (Â°)**	**Left RMSE**	**Right RMSE**
Ti membrane	31.13	33.12	32.13	0.74	0.49
Ti-TiO_2 (10 nm)	141.09	144.29	142.69	0.55	0.56

Ti-TiO_2 (100 nm)	126.64	128.96	127.8	0.50	0.48

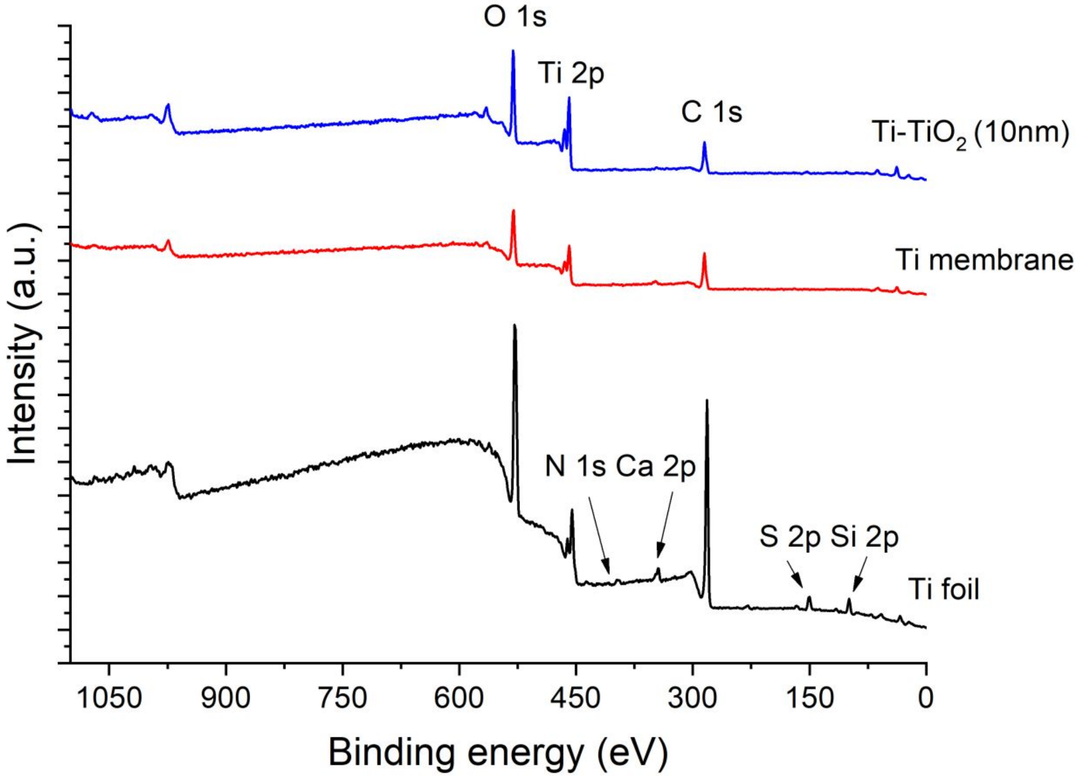

Figure S2. XPS survey spectra of pristine Ti foil, laser-nanostructured Ti surface, and ALD-modified samples.

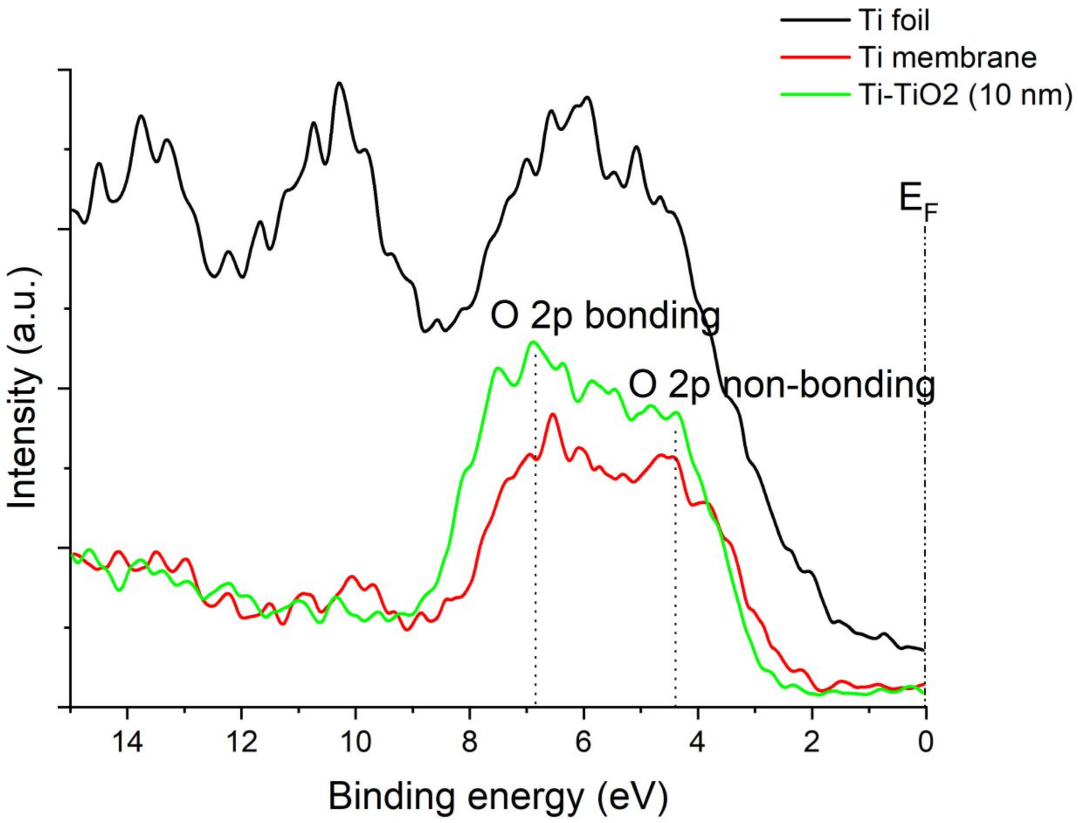

Figure S3. Valence band X-ray photoelectron spectroscopy (XPS) spectra of pristine Ti foil, laser-nanostructured Ti surface, and ALD-modified samples.

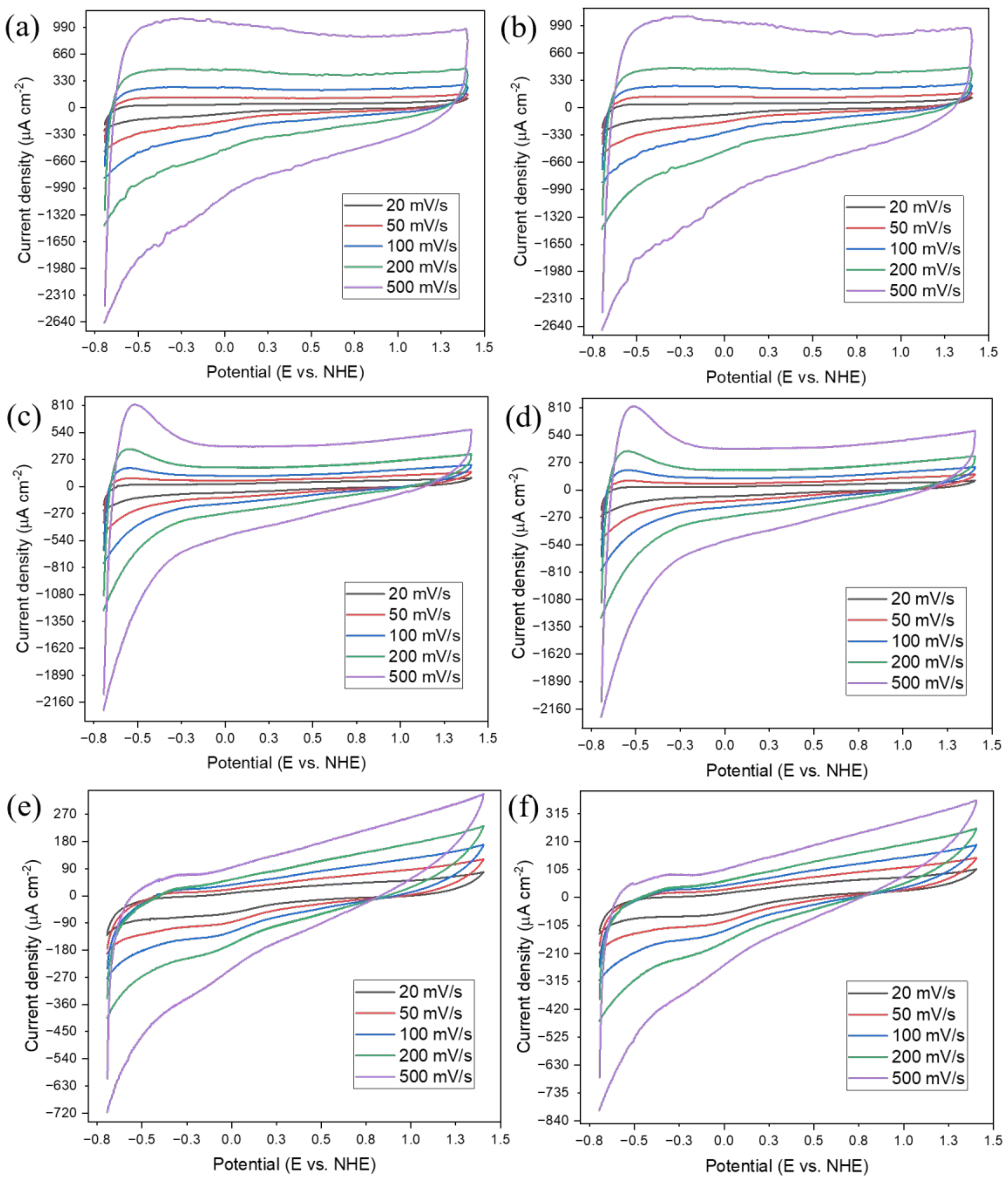

Figure S4. Cyclic voltammetry measurements at various scan rates for (a, b) Ti membrane under dark and illuminated conditions, respectively; (c, d) Ti-TiO_2 (10 nm) under dark and illuminated conditions; and (e, f) Ti-TiO_2 (100 nm) under dark and illuminated conditions.

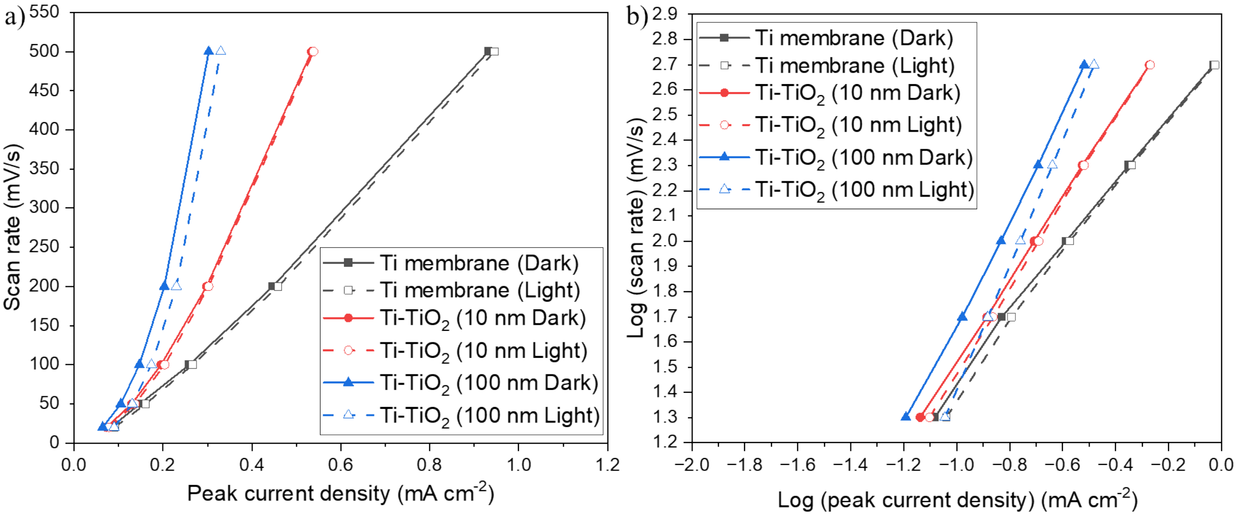

Figure S5. (a) Peak current density versus scan rate and (b) log (peak current density) versus log(scan rate) for all prepared samples.

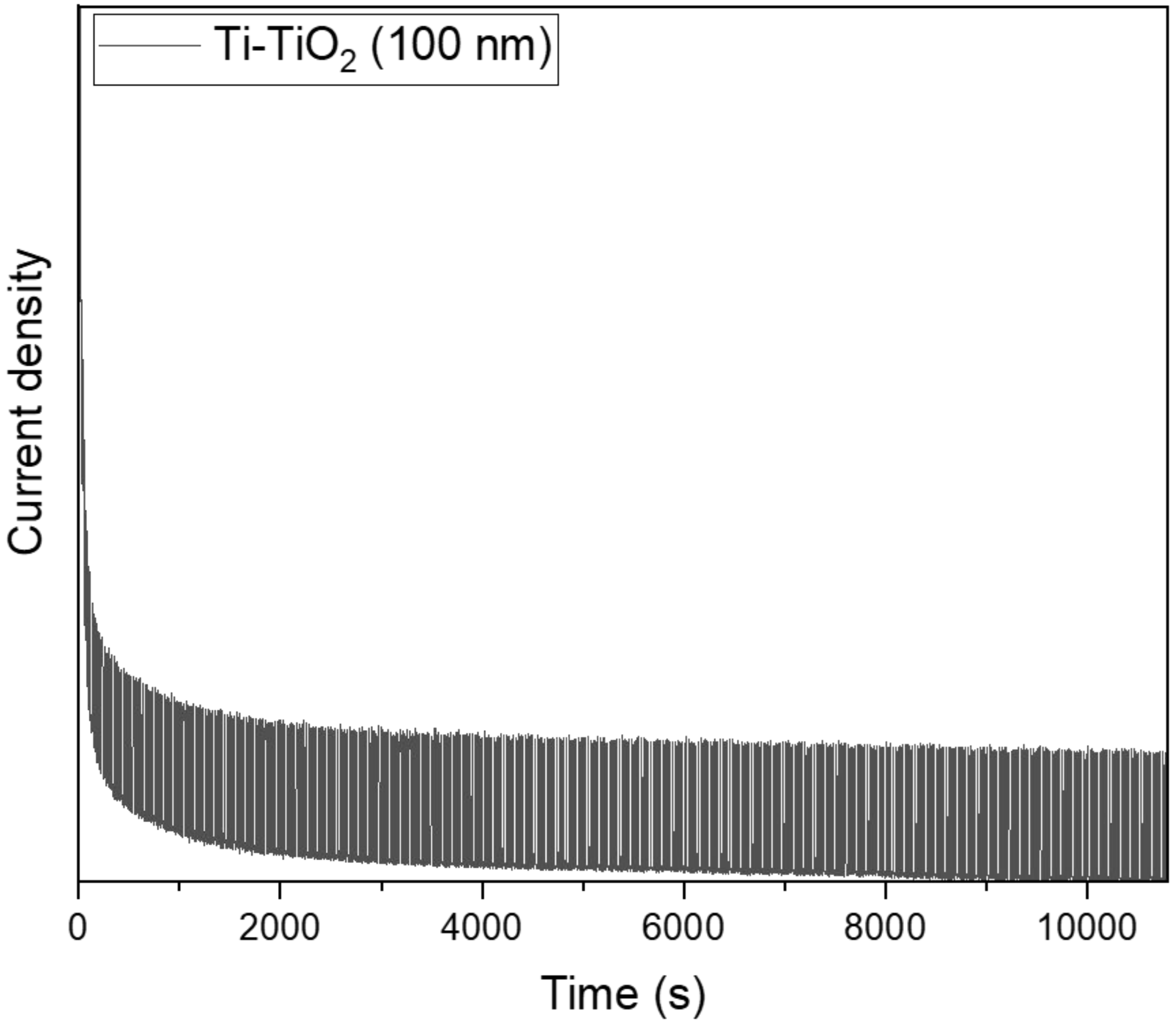

Figure S6. Chronoamperometric stability test with periodic light ON-OFF cycling over 3 hours for the Ti-TiO2 (100 nm) sample.

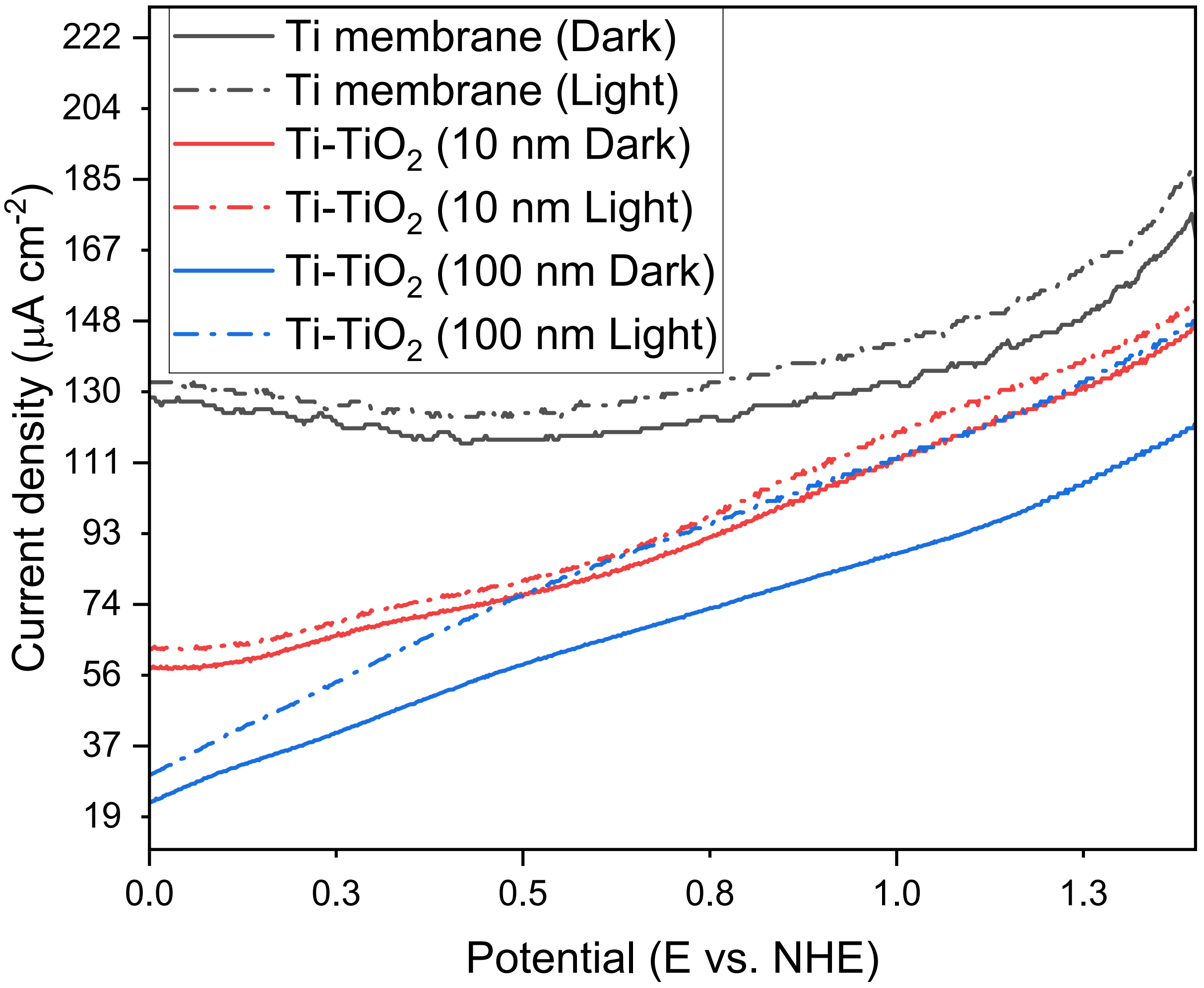

Figure S7. Linear sweep voltammetry measurements of all samples at a scan rate of 50 mV/s

Table S3. Fitted electrochemical impedance spectroscopy (EIS) parameters for the measured samples.

Name	Rs(Ω)	Rct(Ω)	Q (CPE Parameter)	α (CPE Exponent)
Ti membrane (Dark)	14.68	76.17 kΩ	1.02E-03	0.91
Ti membrane (Light)	14.62	73.99 kΩ	1.02E-03	0.91
Ti-TiO_2 (10 nm Dark)	16.66	47.69 kΩ	4.48E-04	0.84
Ti-TiO_2 (10 nm Light)	16.09	52.79 kΩ	4.60E-04	0.83
Ti-TiO_2 (100 nm Dark)	7.68	40.53 kΩ	1.35E-04	0.73
Ti-TiO_2 (100 nm Light)	3.41	39.90 kΩ	1.45E-04	0.71

Table S4. Fitted Mott–Schottky parameters for the measured samples.

**Name**	**V_{fb} (V)**	**N_A (m^{-3})**
Ti membrane (Dark)	-0.44074125	1.44E+19
Ti membrane (Light)	-0.44973125	1.39E+19
Ti-TiO_2 (10 nm Dark)	-0.55784125	3.76E+18
Ti-TiO_2 (10 nm Light)	-0.57120125	3.28E+18
Ti-TiO_2 (100 nm Dark)	-0.44782125	4.34E+19
Ti-TiO_2 (100 nm Light)	-0.48049125	3.63E+19

Table S5. XPS fitting parameters

	Ti foil			Ti membrane			Ti-TiO_2 (10 nm)		
O 1S	**Pos.[eV]**	**FWHM [eV]**	**Area [%]**	**Pos.[eV]**	**FWHM [eV]**	**Area [%]**	**Pos.[eV]**	**FWHM [eV]**	**Area [%]**
O^{2-}	529.67	1.357	33.97	529.6	1.202	67.67	530.04	1.175	75.80
O-H	531.35	1.562	25.38	531.38	1.700	25.91	531.58	1.60	21.1
H_2O	532.39	1.515	40.46	532.80	1.700	6.42	532.80	1.866	3.11
**Ti 2p**	**Pos.[eV]**	**FWHM [eV]**	**Area [%]**	**Pos.[eV]**	**FWHM [eV]**	**Area [%]**	**Pos.[eV]**	**FWHM [eV]**	**Area [%]**
TiO_2 $2p_{3/2}$	458.17	1.165	49.3	458.24	1.128	66.6	458.55	1.149	66.3
TiO_2 $2p_{1/2}$	463.87	2.227	24.55	463.94	2.153	33.4	464.25	2.168	33.4

$Ti_{[A]}$ $2p_{3/2}$	453.89	0.833	14.31						
$Ti_{[A]}$ $2p_{1/2}$	459.69	1.023	7.00						
$Ti_{[B]}$ $2p_{3/2}$	456.51	1.587	3.00						
$Ti_{[B]}$ $2p_{1/2}$	462.01	3	1.50						
**C 1S**	**Pos.[eV]**	**FWHM [eV]**	**Area [%]**	**Pos.[eV]**	**FWHM [eV]**	**Area [%]**	**Pos.[eV]**	**FWHM [eV]**	**Area [%]**
C-C/C-H	284.80	1.20	83.00	284.80	1.17	75.00	284.80	1.35	75.00
C-O	286.24	1.50	12.00	286.04	1.48	20.00	286.39	1.50	20.00
O-C=O	288.51	1.79	5.00	288.29	1.46	5.00	288.63	1.48	5.00

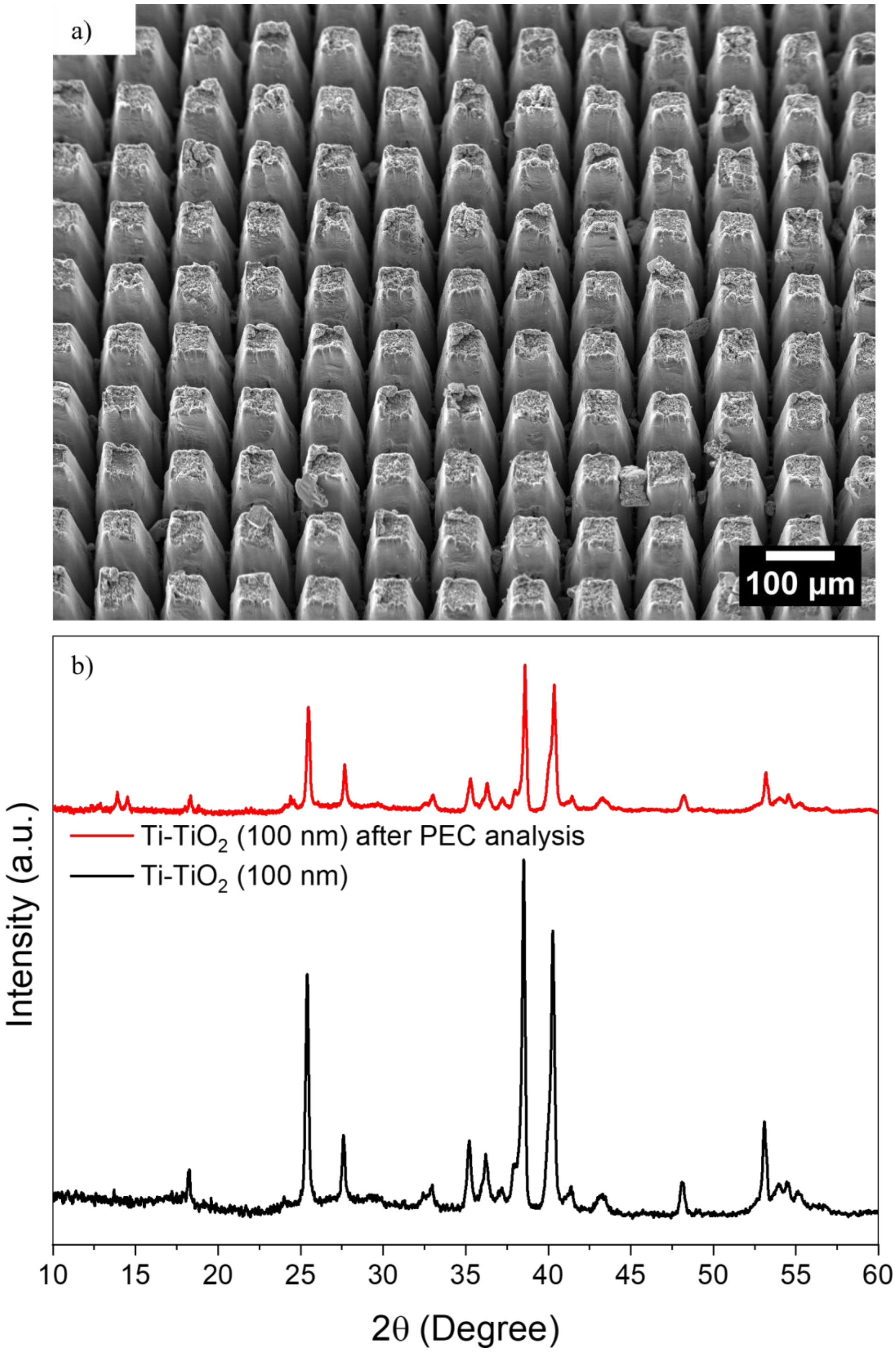

Figure S8. (a) SEM image and (b) XRD pattern of the Ti-TiO2 (100 nm) sample after photoelectrochemical (PEC) testing.
